# Thiazole fused *S*,*N*-heteroacene step-ladder polymeric semiconductors for organic transistors†

**DOI:** 10.1039/d2sc04661j

**Published:** 2022-09-26

**Authors:** Salahuddin Attar, Rui Yang, Zhihui Chen, Xiaozhou Ji, Marc Comí, Sarbajit Banerjee, Lei Fang, Yao Liu, Mohammed Al-Hashimi

**Affiliations:** Department of Chemistry, Texas A&M University at Qatar P.O. Box 23874 Doha Qatar mohammed.al-hashimi@tamu.edu; Beijing Advanced Innovation Center for Soft Matter Science and Engineering, Beijing University of Chemical Technology Beijing 100029 China; Department of Chemistry, Texas A&M University College Station 77843-3255 Texas USA; Department of Chemical Engineering, Stanford University Stanford 94305 California USA

## Abstract

Ladder-type thiazole-fused *S*,*N*-heteroacenes with an extended π-conjugation consisting of six (SN6-Tz) and nine (SN9-Tz) fused aromatic rings have been synthesized and fully characterized. To date, the synthesis of well-defined fused building blocks and polymers of π-conjugated organic compounds based on the thiazole moiety is a considerable synthetic challenge, due to the difficulty in their synthesis. Acceptor–donor building blocks M1 and M2 were successfully polymerized into ladder homopolymers P1–P2 and further copolymerized with a diketopyrrolopyrrole unit to afford step-ladder copolymer P3. The optical, electronic, and thermal properties, in addition to their charge transport behavior in organic thin-film transistors (OTFTs), were investigated. The results showed an interesting effect on the molecular arrangement of the thiazole-based ladder-type heteroacene in the crystal structure revealing skewed π–π-stacking, and expected to possess better p-type semiconducting performance. The polymers all possess good molecular weights and excellent thermal properties. All the polymer-based OTFT devices exhibit annealing temperature dependent performance, and among the polymers P3 exhibits the highest mobility of 0.05 cm^2^ V^−1^ s^−1^.

## Abstract 2

Ladder-type thiazole-fused *S*,*N*-heteroacenes with an extended π-conjugation consisting of six (SN6-Tz) and nine (SN9-Tz) fused aromatic rings have been synthesized and fully characterized.

## Graphical abstract

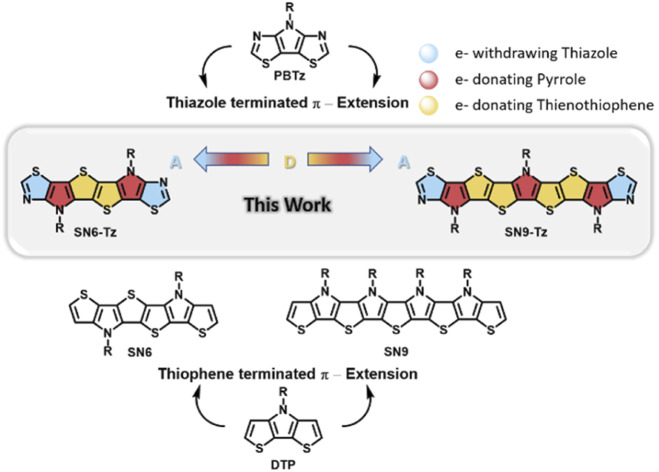

## Introduction

In recent years, there has been a significant drive towards changes in the molecular design and device engineering of semiconductors to optimize their charge-carrier mobilities.^1–3^ Ideally, high mobility can be achieved when polymer units orient themselves in a coplanar configuration favourable for charge delocalization along a π-conjugated backbone.^4,5^ Conjugated ladder-type small molecules and macromolecules, which feature coplanar and rigid π-conjugated backbones, have emerged as an intriguing class of new organic materials, due to their unique electrical, physical and chemical properties.^6–11^ In contrast to conventional conjugated materials which tend to adopt a non-zero dihedral angle conformation as a result of thermal fluctuation or torsional strain,^12^ the fused rings in a π-conjugated ladder-type backbone have a low degree of bond rotation, thus leading to a linear and torsion-free planar conformation. As a result, this leads to the reduction of the re-organizational energy during charge transfer and promotes π–π electron delocalization, enabling materials with higher charge-carrier mobilities in the condensed phase.^13–15^ Several examples of such conjugated ladder-type copolymers with impressive high mobilities are composed of donor or acceptors units including pentacyclic indacenodithiophene (IDT),^16–19^ indacenodiselenophene (IDSe),^20^ indacenodithiazole (IDTz),^21^ and bithiophene imide (BTI).^22^

**Fig. 1:**
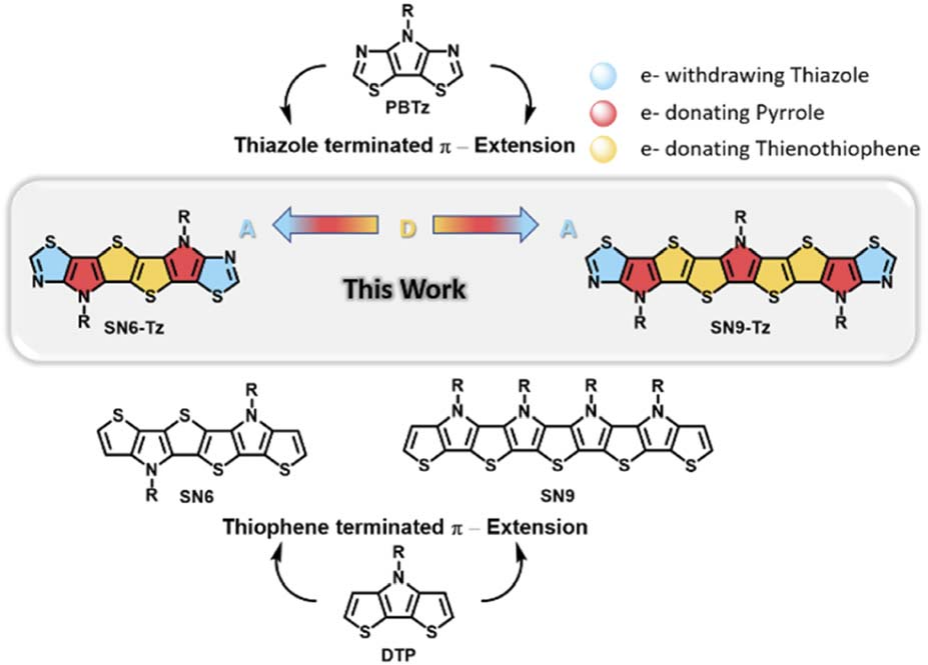
Chemical structures of relevant PBTz, DTP, and *S*,*N*-heteroacene ladder building blocks and the newly synthesized thiazole fused SN6-Tz and SN9-Tz ladder moieties.

Another interesting class of fused ladder-type building blocks with promising electronic and optical properties are based on heteroacenes.^23–34^ Among the various reported building blocks, *S*,*N*-heteroacenes consisting of fused thiophene and pyrrole rings, a structural analogue of the electron-donating dithieno[3,2-*b*:2′,3′-*d*]pyrrole (DTP), unit are of particular interest.^35–38^ Introducing solubilizing substituents on the sp^2^-hybridized nitrogen atom of the pyrrole moiety, enhancing the intramolecular charge transfer (ICT) interactions, and tuning the energy levels are promising strategies for influencing the properties required to achieve high performance organic thin-film transistors (OTFTs). Mitsudo *et al.* reported the first example of a fused aromatic *S*,*N*-heteroacene (SN5) consisting of five membered-rings. The highest occupied molecular orbital (HOMO) and the lowest unoccupied molecular orbital (LUMO) energy levels of the small molecules were tuned by the functional groups on the nitrogen atoms.^39^ Extending the structure to six-fused aromatic rings afforded *S*,*N*-heterohexacene (SN6) (Fig. 1), which was reported by Bäuerle and co-workers.^40^ Vacuum-deposited films of the acceptor-capped SN6 oligomers exhibited a mobility of 0.021 cm^2^ V^−1^ s^−1^. The preparation of *S*,*N*-heteroacenes was further extended from SN8 to a stable SN13 tridecamer, providing data with an interesting structure–property relationship.^41,42^ Most recently, Wong and co-workers reported the synthesis of donor–acceptor (D–A) alternating copolymers containing pentacyclic *S*,*N*-heteroacene building blocks. OTFT devices fabricated using the step-ladder copolymer exhibited a hole mobility of 0.1 cm^2^ V s^−1^.^43^

However, all current reported examples are based on *S*,*N*-heteroacenes consisting of fused thiophene and pyrrole units in the backbone. Replacing the flanked thiophene units with the more electron-deficient thiazole moieties is a promising approach to increase the ionization potential and improve the oxidative stability. Such an example as depicted in Fig. 1 includes fused pyrrolo[3,2-*d*:4,5-*d*′]-bisthiazole (PBTz).^34,44–46^ In addition, the flanking thiazole units can provide potential anchoring points for the formation of non-covalent bonds or can enhance the van der Waals interaction with neighbouring units to further rigidify and coplanarize the conjugated system.^47^ Until now there have been no reported examples of thiazole-fused *S*,*N*-heteroacene building blocks, which can be attributed to the difficulty in their synthesis.

Herein, we report the first example of ladder-type thiazole-fused *S*,*N*-heteroacenes with an extended π-conjugation in the backbones consisting of six and nine fused aromatic rings (Fig. 1). The acceptor–donor building blocks were successfully polymerized into ladder homopolymers and copolymerized with a diketopyrrolopyrrole (DPP) unit to afford a step-ladder copolymer. Their optical, electronic, and thermal properties and charge transport behavior in OTFTs were investigated. The results showed an effect on the molecular arrangement of the thiazole-based ladder-type heteroacene in the crystal structure revealing a skewed π–π-stacking at a higher order. The polymer-based OTFT devices exhibit annealing temperature dependent performance with the highest mobility of 0.05 cm^2^ V^−1^ s^−1^ obtained for P3.

## Results and discussion

### Monomer synthesis

**Scheme 1:**
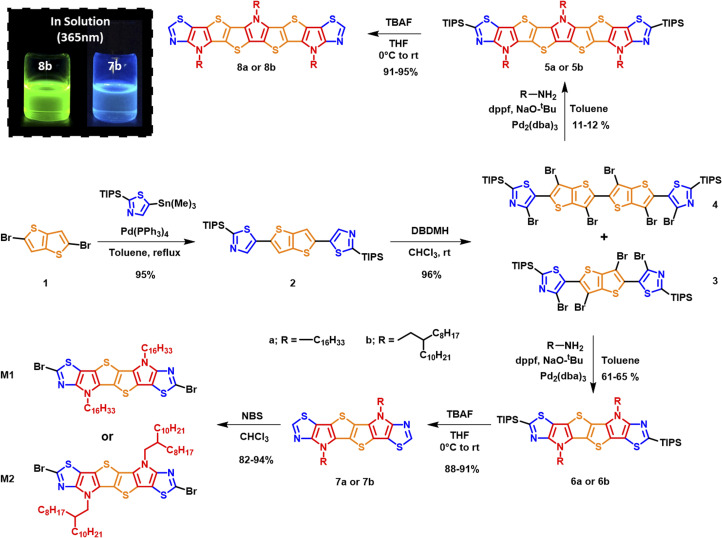
Synthesis of SN6 heteroacene monomers M1 and M2 and SN9 monomers 8a and b.

The synthetic route to monomers M1 and M2 is depicted in Scheme 1. Stille coupling of 2-(triisopropylsilyl)-5-(trimethylstannyl)thiazole with 2,5-dibromothieno[3,2-*b*]thiophene 1 afforded 2,5-bis(2-(triisopropylsilyl)thiazol-5-yl)thieno[3,2-*b*]thiophene 2 as an off-yellow solid in 95% yield. The structure of 2 was confirmed using single crystal X-ray diffraction (XRD) (Fig. S1),† and the bond lengths and torsion angles are summarized in Tables S1 and S3.† The subsequent bromination of compound 2 using excess 1,3-dibromo-5,5-dimethylhydantoin (DBDMH) yielded compound 3 as a bright yellow solid. In addition, the reaction also afforded 4 in small amounts as a side product.

**Fig. 2:**
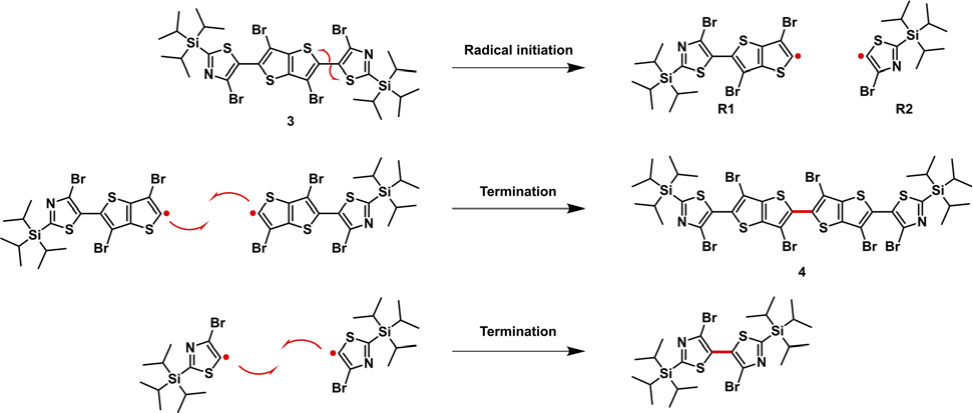
Postulated mechanism for the formation of monomer 4.

**Fig. 3:**
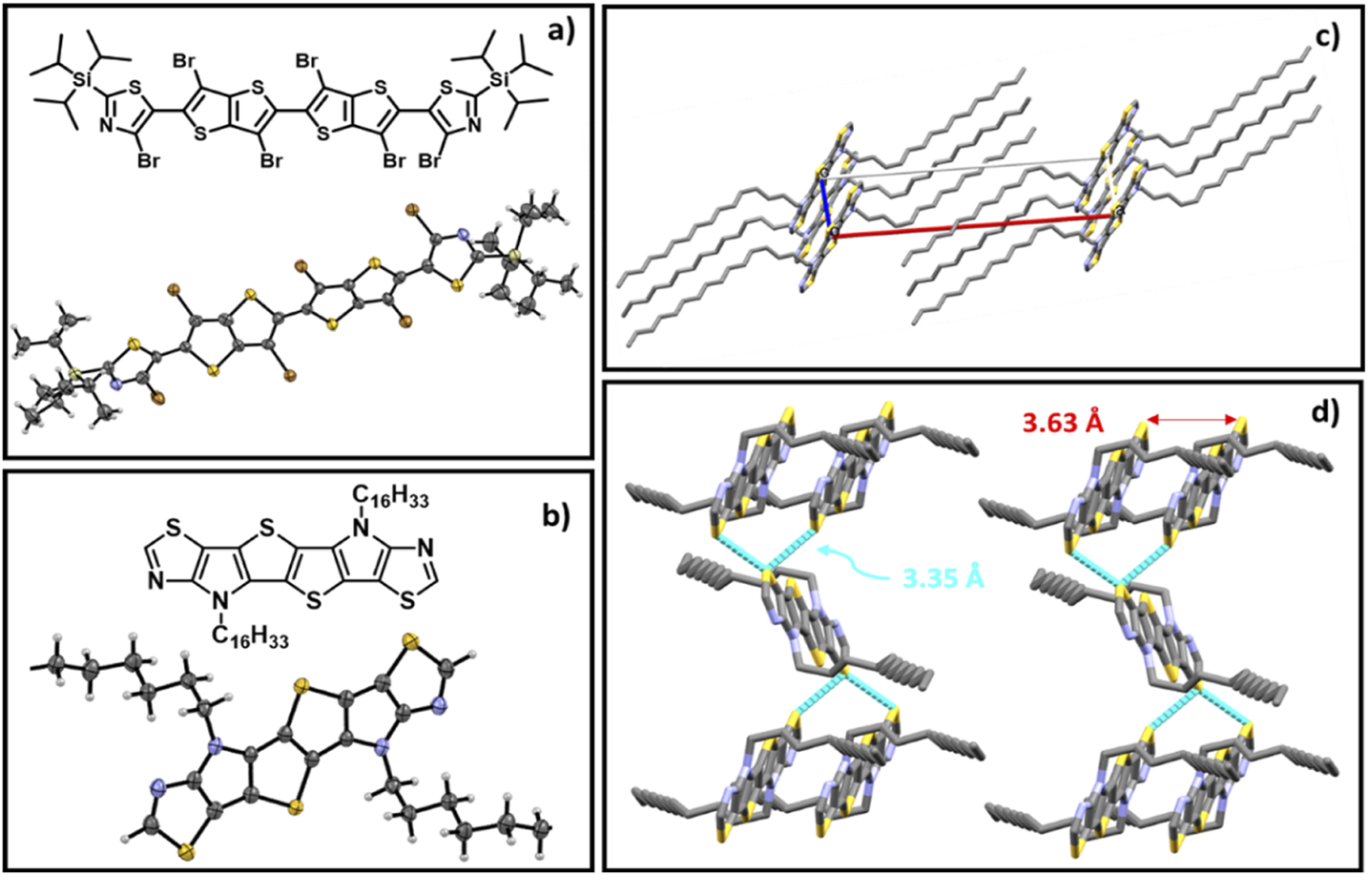
Molecular structures of (a) monomer 4; (b) 7a (R = *n*-hexadecyl) with thermal ellipsoids drawn at the 50% probability level; (c) crystal packing of 7a along the *b*-axis; (d) packing of 7a showing the distance of S–S interactions (displayed in turquoise blue), and the stacking distance (shown in red) where H atoms are omitted for clarity.

We postulate that the formation of 4 can be a result of a radical initiation that takes place by the homolytic fission of monomer 3 into R1 and R2 radicals (Fig. 2). The radical R1 then can either recombine with R2 to generate monomer 3 or react with itself to form 4, and this was only confirmed once the sample was characterized using single crystal XRD analysis (Fig. 3a,Tables S4 and S5).† In addition, the homo-dimer of R2 can also be observed in small traces as evaluated by NMR spectroscopy.

**Scheme 2:**
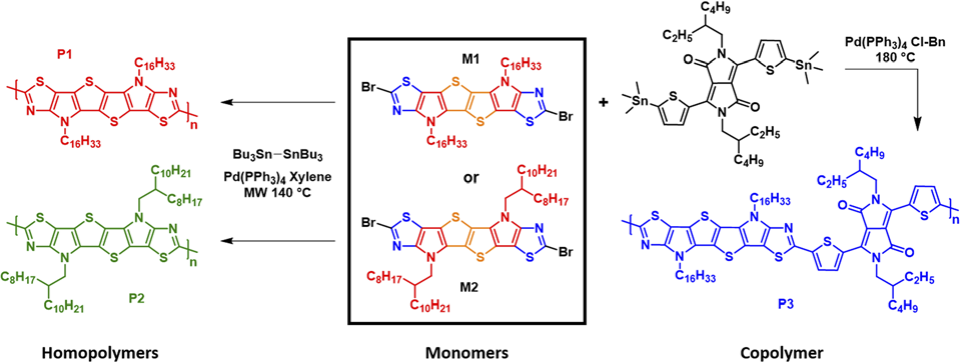
Synthesis of homopolymers P1 and P2 and copolymer P3.

Buchwald–Hartwig amination of the crude precursor 3 containing trace amounts of 4 (used without further purifications) with hexadecyl amine or 2-*n*-octyl-1-dodecylamine in a sealed microwave vial at 100 °C in the presence of tris(dibenzylideneacetone)dipalladium(0) as the catalyst under basic conditions afforded triisopropylsilyl (TIPS) protected six membered fused ladder monomers 6a and 6b, and nine-membered ladder monomers 5a and 5b from precursor 4. Subsequently, TIPS deprotection of monomers 5a/b and 6a/b with TBAF afforded 7a/b and 8a/b in good overall yield. Initial attempts to dibrominate monomers 7a and 7b using *N*-bromosuccinimide (NBS) at room temperature did not yield the desired products. Successful bromination using NBS at a lower temperature (−15 °C) in anhydrous chloroform afforded the desired monomers M1 and M2 (Schemes 1 and 2) in 94% and 82% yield, respectively.

Interestingly the six thiazole fused *S*,*N*-heteroacene monomer 7b exhibits a blue emission in chloroform solution under 365 nm UV light, while the nine-heterocyclic ring system 8b exhibits green emission (Scheme 1), which can be attributed to the extension of the heterocyclic ring system. Single crystal XRD analysis confirmed the structure of the hexadecyl ladder monomer 7a (Fig. 3b, Tables S6 and S7),† which crystallizes in the monoclinic crystal system, exhibiting a centrosymmetric space group of *P*2_1_/*c* at the point of inversion. In comparison to the thiophene-fused SN6 single crystal structure reported by Bäuerle,^40^ which has two equivalent molecules in the unit cell, the thiazole-fused 7a also exhibits two equivalent molecules in the unit cell, with a 2-fold screw axis of symmetry; *a* = 25.4850(5), *b* = 14.7657(3), and *c* = 5.59660(10) Å; *α* = 90.00, *β* = 94.742 (2), and *γ* = 90.00° (additional information such as bond lengths and torsion angles is summarized in Tables S6 and S7).† Interestingly, the ordered non-covalent self-assembly established in the crystal packing of the thiophene based system SN6 was reported to be 3.37 Å, with a π–π interaction at a distance of 3.55 Å.^40^ However, the thiazole containing moiety 7a along the *c*-axis through the flanking thiazole sulfur–sulfur (S–S) interactions was measured to have a slightly shorter contact of 3.35 Å, which is lower than the sum of van der Waals radii (1.8 Å for one S) (Fig. 3d). The molecular arrangement of 7a in the crystal structure reveals a skewed π–π stacking with a distance of 3.63 Å in the columns. The bond angle of S–C–N in the thiazole moiety is 116.6°, while for the thiophene fused SN6 it was reported to be 113.8° which is slightly lower. The presence of the nitrogen atom in the thiazole moiety also has an effect on the bond length (1.305 Å) in comparison to the thiophene counterpart (1.366 Å) in SN6. Intermolecular charge transfer is closely related to the orientation and molecular packing in the backbone. Based on the single crystal structure of 7a, charge transfer integrals between two π–π stacked molecules were calculated. A large hole charge transfer integral between two molecules is found at 0.27 eV, which indicates efficient hole transfer in the single crystal. However, the electron transfer is less efficient with a transfer integral at 0.13 eV, and thus, the polymers derived from thiazole-fused *S*,*N*-heteroacenes are expected to possess better p-type semiconducting performance. The good intermolecular charge transfer could be attributed to the co-facial orientation between molecules, which is promoted by the ladder-type backbone structure. As a comparison, the calculated hole and electron charge transfer integrals in compound 2 are lower (0.08 and 0.03 eV) than that of 7a, 0.27 and 0.12 eV, respectively. This can be attributed to the twisted stacking orientation present in the molecule (Fig. S2 and Table S8†).

### Polymer synthesis

As depicted in Scheme 2 homopolymers P1 and P2 were prepared *via* microwave-assisted Stille coupling of M1 and M2 with bis(tributyltin) in xylene using tetrakis(triphenylphosphine)palladium(0). Copolymer P3 was synthesized by copolymerization of monomer M1 with the stannylated DPP monomer. All the polymers were precipitated in acidified methanol and purified *via* Soxhlet extraction with a sequence of refluxing methanol, acetone, and *n*-hexane. Finally refluxing chloroform was used to extract the polymers. After removing the solvent, homopolymers P1 and P2 were isolated as dark blue solids, while the copolymer P3 was isolated as a dark green solid. All the polymers possessed good solubility in common chlorinated solvents such as chloroform and chlorobenzene. The number-average molecular weight (*M*_n_) and the polydispersity index (*Đ*) were determined *via* gel permeation chromatography (GPC) in chlorobenzene solution at 85 °C using polystyrene standards as the calibrants. The *M*_n_ of homopolymers P1 and P2 was measured to be 11.7 and 13.6 kDa, respectively, with a narrow *Đ* in the range of 1.8–2.0, while copolymer P3 exhibited an *M*_n_ of 20.1 kDa with a *Đ* of 2.1. Thermogravimetric analysis (TGA) and differential scanning calorimetry (DSC) under an inert atmosphere were used to investigated the thermal properties of polymers P1–P3 (Fig. S3a and b).† The thermal decomposition temperatures (*T*_d_) at 5% weight loss for the three polymers P1–P3 were measured to be above 300 °C. Homopolymers P1 and P2 having different alkyl side chains (linear *vs.* branched) and the copolymer P3 presented similar features with the onset weight loss corresponding to the elimination of alkyl side chains. The DSC data did not reveal any pronounced thermal transitions in the range of 25 to 300 °C, as a result of the low degree of order in the polymer thin film.

### Optical and electrochemical properties

**Fig. 4:**
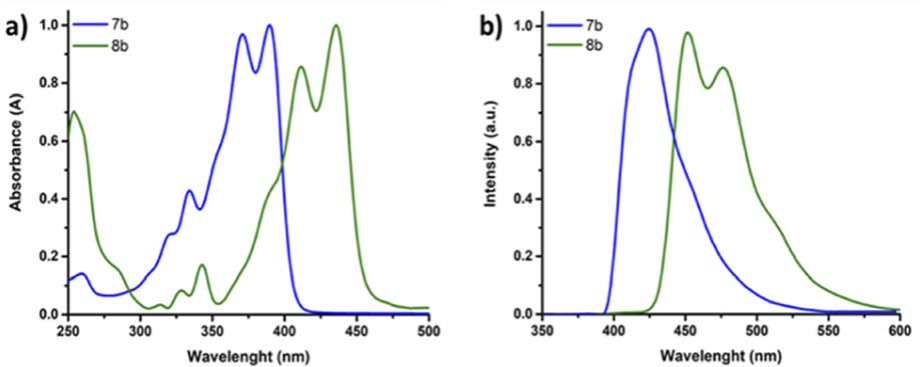
(a) Normalized UV-vis absorption and (b) emission spectra of SN6-Tz oligomer 7b (blue) and SN9-Tz oligomer 8b (green) in chloroform solution.

The optical properties of the novel thiazole fused heteroacene SN6-Tz7b and SN9-Tz8b monomers were investigated using Ultraviolet-visible (UV-vis) and fluorescence spectroscopy as depicted in Fig. 4 and summarized in Table 1. Typically, multiple vibronic splitting absorption bands are observed in *S*,*N*-heteroacenes, and this is due to the rigidity of the highly ordered, planar and fused molecular π-backbone. SN6-Tz (7b) exhibits vibronically resolved absorption bands (Fig. 4a), at *λ*_max_ = 334 and 321 nm corresponding to π–π* electronic transitions perpendicular to the molecular axis and an energy band at *λ*_max_ = 371 and 390 nm attributed to the π–π* electronic transitions along the molecular axis. The lower energy transitions correspond to the HOMO–LUMO energy gap in the conjugated π-system.^42,48^ In comparison, the thiophene fused SN6 monomer exhibited energy absorption bands at *λ*_max_ = 362 and 380 nm. Thus, replacing the thiophene units with the thiazoles in the ladder moiety slightly lowers the optical band gap from *E*g^opt^ = 3.12 eV in SN6 to 3.03 eV in SN6-Tz (7b). SN6-Tz with an A–D–A architecture is expected to result in a smaller band gap in comparison to the SN6 counterpart. However, the polarized nature of the thiazole ring makes the heterocyclic thiazole unit act as both a donor and an acceptor.^49^ The stronger electron-donating ability of the two neighbouring substituents makes SN6-Tz a weaker D–A than expected, and as a result only a small difference of ∼0.12 eV is seen in the band gap. As depicted in Fig. 4a when increasing the conjugation length from six units in SN6-Tz (7b) to SN9-Tz (8b) the absorptions bands are significantly red shifted by 41–46 nm (*λ*_max_ = 412 and 436 nm), thus lowering the optical band gap further. Increasing the aromatic fused units from three in the DTP moiety (SN3), to five (SN5), six (SN6), and nine (SN9) fused heterocycles, or even in the case of the thiazole moieties SN6-Tz and SN9-Tz leads to the main absorption maximum to gradually red-shift. On exciting SN6-Tz at 335 nm, a strong emission at 425 nm was observed (Fig. 4b), which is slightly red shifted in comparison to the thiophene SN6 (411 nm) counterpart. The thiazole fused heteroacene SN6-Tz (7b) displayed a slightly higher Stokes shift of 2111 cm^−1^ compared to its thiophene fused SN6 counterpart (1985 cm^−1^), due to a higher dipole moment present in the excited state as a result of the electron withdrawing effect of thiazole units present at the peripherals. Similar results were observed for the extended nine membered SN9-Tz (8b) ladder monomer, exhibiting strong emission at 451 nm, with a broad shoulder at 490 nm. In addition, SN9-Tz (8b) also showed a Stokes shift of 792 cm^−1^.

**Table 1:** Optical properties of thiazole fused *S*,*N*-heteroacene monomers 7b and 8b determined by UV-vis absorption and emission spectroscopy

*S*,*N*-Heteroacene	*λ*_max_ (Abs)^a^ [nm]	*λ*_max_ (*E*_m_)^a^ [nm]	Stokes shift (Δ*E*_s_)^b^ [cm^−1^]	*E*g^opt^^c^ [eV]	*E*_HOMO_ d [eV]	*E*_LUMO_ d [eV]
SN6	380	411	898	3.15	−4.85	−1.24
SN6-Tz (7b)	390	425	2111	3.03	−5.19	−1.58
SN9	—	—	—	—	−4.66	−1.53
SN9-Tz (8b)	436	451	762	2.70	−4.85	−1.76

a Measured in CHCl_3_.

b Stokes shift-difference between the 0–0 vibronic transitions of the absorption and emission spectra.

c Calculated from the low-energy onset of the absorption band.

d Determined by the DFT method.

**Fig. 5:**
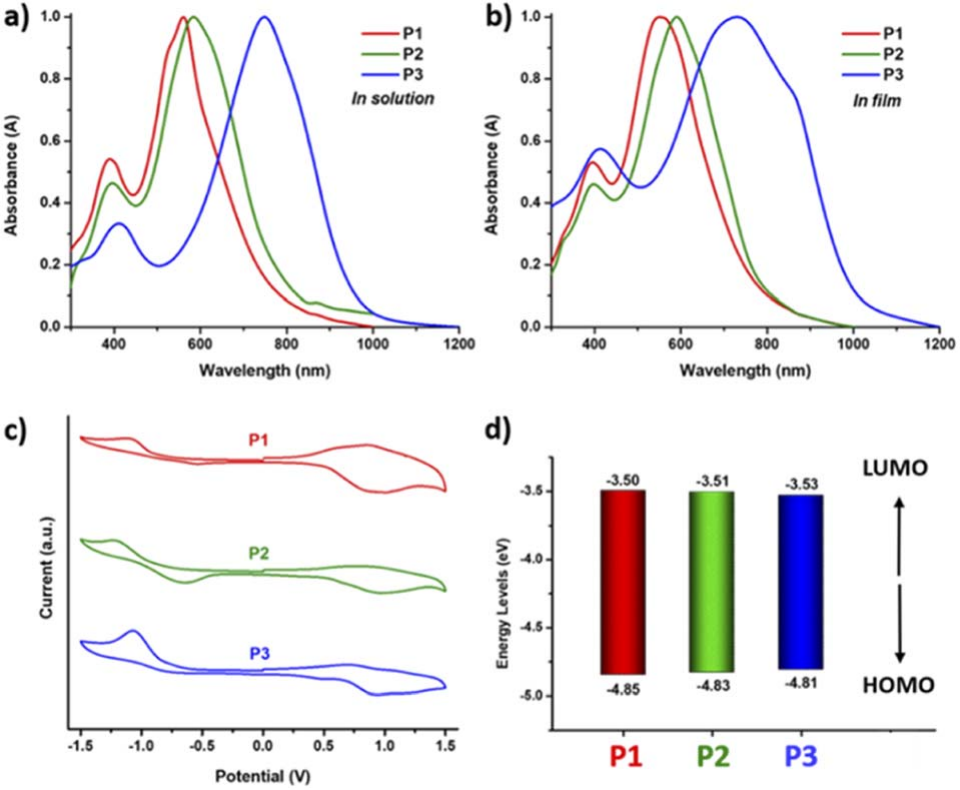
Characterization of polymers P1, P2 and P3 (a) UV-vis absorption spectra in chloroform solution; (b) as a thin film. (c) cyclic voltammograms as a thin film (scan rate 100 mV s^−1^) and (d) HOMO–LUMO energy level diagram obtained using CV measurement.

The UV-vis absorption spectra of polymers P1–P3 in chloroform solution and thin films are depicted in Fig. 5 and the optical and electrochemical properties are summarized in Table 1. Both homopolymers P1 and P2 in solution display a HOMO–LUMO absorption band represented by π–π* transitions in the visible region peaking at around 560 to 580 nm, while the n–π* energy bands are observed at around 400 nm. By incorporating the strong accepting DPP unit into the copolymer P3, the LUMO energy level is lowered and the absorption peak is red-shifted to 750 nm.

**Table 2:** Parameters of field-effect transistor devices based on polymers P1–P3. *Top-gate/bottom contact (TG/BC)

Material	Method	*μ*_ave_ (cm^2^ V^−1^ s^−1^)^a^	*μ*_max_ (cm^2^ V^−1^ s^−1^)^a^	*V* ^th^ (V)	*I*_On_/*I*_Off_
P1	RT	1.6 × 10^−4^	3.2 × 10^−4^	11/7	10
	**200 °C**	**1.3 × 10** ^**−3**^	**3.1 × 10** ^**−3**^	**−3/−8**	**10** ^**3**^
	250 °C	2.2 × 10^−4^	2.7 × 10^−4^	−9/16	10^3^
	300 °C	1.2 × 10^−4^	2.0 × 10^−4^	−12/−16	10^2^
P2	RT	6.1 × 10^−5^	1.3 × 10^−4^	−4/−8	10^2^
	120 °C	7.7 × 10^−5^	2.5 × 10^−4^	−9/−17	10^3^
	180 °C	3.0 × 10^−5^	6.6 × 10^−5^	−14/−16	10^3^
	**200 °C**	**2.0 × 10** ^**−4**^	**4.5 × 10** ^**−4**^	**−8/−15**	**10** ^**3**^
P3	RT	1.4 × 10^−4^	2.2 × 10^−4^	−20/−23	10^3^
	120 °C	8.2 × 10^−4^	1.6 × 10^−3^	−25/−29	10^3^
	180 °C	1.2 × 10^−3^	2.4 × 10^−3^	−14/−26	10^3^
	200 °C	3.0 × 10^−3^	3.3 × 10^−3^	−21/−27	10^4^
	*200 °C	**0.04**	**0.05**	**−23/−27**	**10** ^**3**^
	250 °C	1.7 × 10^−3^	2.2 × 10^−3^	−23/−28	10^4^
	300 °C	6.4 × 10^−4^	7.7 × 10^−4^	−23/−28	10^3^

a Determined from the following equation in the saturation region; *I*_DS_ = (*W*/2*L*) *C*iμ (V_GS_ – V_th_)^2^.

Going from solution to the solid state, P1 shows a slight blue shift and broadening of the maximum absorbance peak (*λ*_max_) from 561 nm to 550 nm, while P2 shows a slight red shift from 584 nm to 592 nm, indicating the different optical behaviour of the polymer as a result of side chain engineering. The copolymer P3 shows a blue shift from 750 to 745 nm with broadening of the absorption and a pronounced shoulder peak visible around 855 nm, and this can be attributed to aggregation with strong interchain π–π stacking, which is beneficial for regular structural organization of copolymer backbones in the solid state.^50–52^

The optical band gaps (*E*g^opt^) of P1 and P2 calculated from the onsets of the absorption spectra in the thin films are 1.49 and 1.51 eV, while the absorption onset of P3 is 1030 nm, resulting in a lower *E*g^opt^ of 1.22 eV. Cyclic voltammetry (CV) was used to measure the oxidation and reduction potentials of polymers P1−P3 in anhydrous acetonitrile under a nitrogen atmosphere. The HOMO and LUMO energy levels of the polymers were calculated using reduction and oxidation peaks with onset potentials relative to the ferrocene/ferrocenium (Fc/Fc^+^) redox potential as shown in Fig. 5c. All the polymers show distinct oxidation and reduction bands indicating both electron donating and electron accepting characteristics. Homopolymers P1 and P2 exhibit distinct quasi-reversible oxidation bands with onset potentials (*E*_ox_) at 0.51 and 0.49 V and an irreversible reduction band with onset potentials (*E*_red_) at −0.84 and −0.83 V, respectively. The calculated HOMO/LUMO energy levels of P1 and P2 are −4.85/−3.50 eV and −4.83/−3.51 eV. In comparison, copolymer P3 presented strong irreversible oxidation and reduction bands, with *E*_ox_/*E*_red_ at 0.47/−0.81 V, which correspond to HOMO/LUMO energy levels at −4.81/−3.53 eV. The estimated electrochemical band gaps, E^el^_g_, of polymers P1–P3 are in the range of 1.28–1.35 eV and are consistent with the *E*g^opt^ calculated from the UV-vis absorption spectra.

### DFT computational studies

**Fig. 6:**
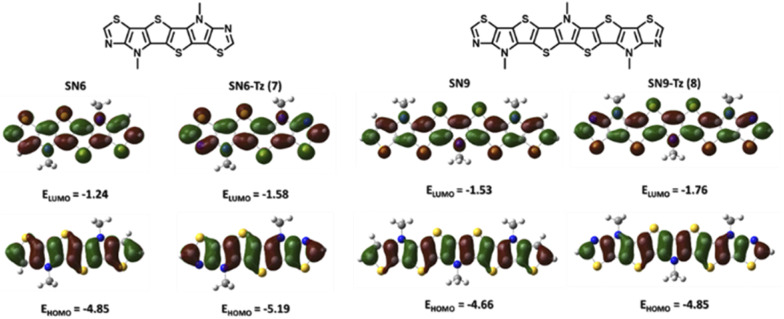
Optimized geometries of thiophene fused SN6, thiazole fused SN6-Tz, thiophene fused SN9 and thiazole fused SN9-Tz molecules and spatial electron distributions of frontier molecular orbitals (FMOs) and their energy levels.

To evaluate the effect of replacing the fused thiophene with the fused thiazole unit, a comparative DFT calculation (B3LYP/6-311G+(d,p)) was performed based on the structures of SN6 and SN6-Tz. Structure optimization was performed at the same level for SN9 and SN9-Tz, while all alkyl groups were simplified with methyl groups to shorten calculation times. Compared to the thiophene end groups in SN6 and SN9, the electron withdrawing thiazole units show slightly lower electron density (Fig. 6) on the HOMO and higher electron density on the LUMO, and thus as a result decrease both HOMO and LUMO energy levels. The thiazole groups pull the electron density from the heterocyclic SN-π-system, leading to a donor–acceptor system, and thus the stabilization of the frontier molecular orbital energies is achieved with narrowing of the band gaps. The extended SN9-Tz heteroacene shows an obviously lower calculated energy band gap (3.09 eV) than SN6-Tz (3.61 eV). However, the calculated results are both slightly at a higher end than the experimental *E*g^opt^ values; this could be attributed to the solvation effect.

### OTFT charge transport characterization

The charge transport properties and electrical behavior of P1–P3 polymers were analysed by fabricating bottom-gate/bottom-contact (BG/BC) OTFTs (Table 2). Gold (Au) and titanium (Ti) layers were thermally evaporated onto a SiO_2_ (300 nm)/p++-Si substrate using a shadow mask to form source and drain contacts, and this was followed by octadecyl trimethoxy silane (OTS) treatment.

**Fig. 7:**
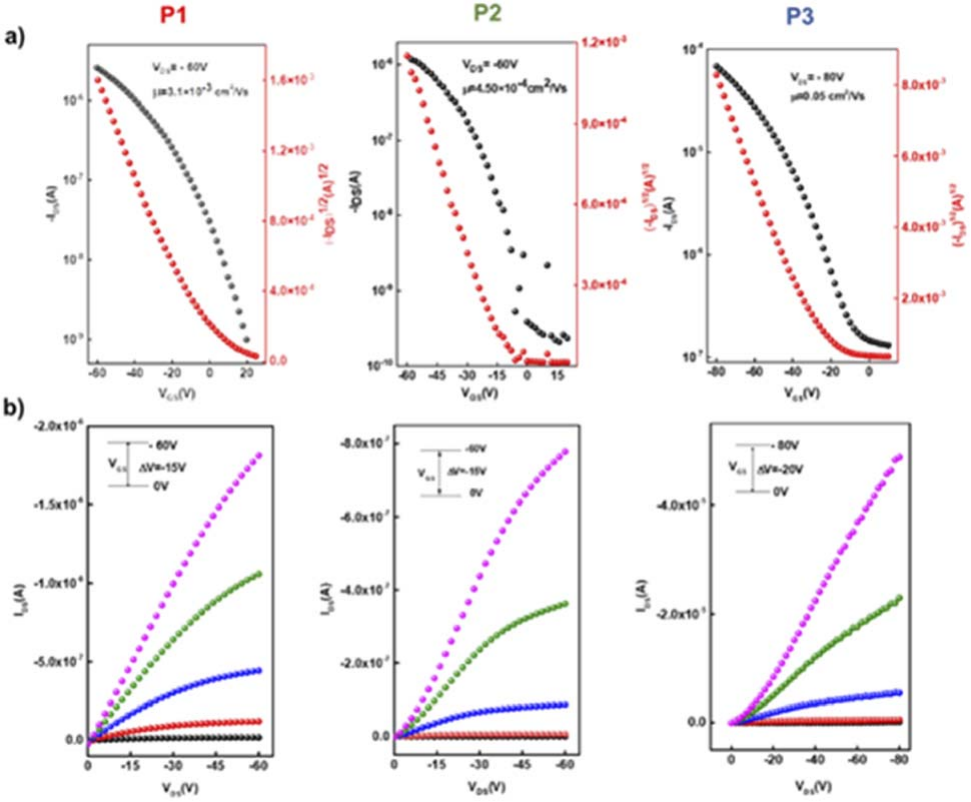
(a) Transfer and (b) output characteristics of the best OTFT devices based on polymers P1, P2, and P3 under optimal conditions.

The polymer solutions were prepared with the same concentration of 5 mg mL^−1^ in chloroform and were coated on the OTS-treated substrate to form organic semiconducting layers in a N_2_-filled glovebox using spin-coating and then annealed at different temperatures for 15 min. The typical output and transport characteristics for the devices based on P1–P3 are depicted in Fig. 7 and ESI.† The charge carrier mobilities (μ) and the threshold voltage (*V*^th^) were extracted from the saturated region in the transfer curves. The OTFT optimal polymer device performance is summarized in Table 3. The initial fabrication of BG/BC devices at various annealing temperatures in order to optimize the charge mobility of polymers P1–P3 was carried out. Generally, thermal annealing represents a satisfactory strategy to optimize the device performance *via* improving film formation and promoting the “edge on” orientation and crystallinity of polymer chains, which are believed to benefit the charge mobility.^53,54^ Interestingly, the mobility performance of all polymers exhibits annealing-temperature dependence up to 200 °C, with better improvements in the transistor performance for homopolymer P1 and copolymer P3, which contain a straight chain substituted ladder unit. This indicates that having a linear alkyl substituent on the pyrrole fused ring can improve the polymer skeleton arrangement with increasing annealing temperature.^55^ Without annealing, the devices fabricated for the homopolymers displayed a maximum charge mobility of 3.2 × 10^−4^ cm^2^ V^−1^ s^−1^ for P1 and 1.3 × 10^−4^ cm^2^ V^−1^ s^−1^ for P2, while copolymer P3 presented a maximum mobility of 2.2 × 10^−4^ cm^2^ V^−1^ s^−1^. After thermal annealing at 200 °C for 15 min, the mobilities were improved by one order of magnitude for the linear alkyl substituted ladder unit-based polymers P1 and P3, with charge mobilities ranging from 3.1–3.3 × 10^−3^ cm^2^ V^−1^ s^−1^. Devices fabricated from homopolymer P2 exhibit discrete fluctuations of charge mobility with increasing thermal annealing temperatures with the best performance of 4.5 × 10^−4^ cm^2^ V^−1^ s^−1^ obtained at 200 °C. In addition, thermal annealing at higher temperatures of 250 and 300 °C for devices based on P1 and P3 resulted in lower charge carrier mobility by one order of magnitude of 2.0 × 10^−4^ and 7.7 × 10^−4^ cm^2^ V^−1^ s^−1^. We further attempted to enhance the OTFT performance of copolymer^56^P3 by exchanging the configuration of the device to top-gate/bottom contact (TG/BC). After annealing at 200 °C, P3 displayed an improved mobility of 0.05 cm^2^ V^−1^ s^−1^ and a *V*^th^ of −23 V, with an on/off current ratio of 10^3^. Meanwhile no significant change was observed for the other polymers P1 and P2 using the TG/BC device architecture.

**Table 3:** Summary of the molecular weights, thermal, optical, electrochemical, XRD and AFM parameters of polymers P1–P3

	*M*_n_ a (kDa)	*Đ*	*T*_d_ (°C)	*λ*max^sol^^b^ (nm)	*λ*max^film^^c^ (nm)	HOMO^d^ (eV)	LUMO^d^ (eV)	(E^el^_g_)^e^ (eV)	*E*g^opt^^f^ (eV)	Lamellar *d*^dd^^,^^g^ (Å)	π–π *d*^dd^^,^^g^ (Å)	RMS (nm)
P1	12	1.8	314	561	550	−4.85	−3.50	1.35	1.51	21.53	3.77	0.80
P2	14	2.0	324	584	592	−4.83	−3.51	1.32	1.49	23.86	4.50	1.04
P3	20	2.1	326	750	745	−4.81	−3.53	1.28	1.22	19.19	3.69	0.75

a Determined by GPC (against polystyrene standards) in chlorobenzene at 85 °C.

b *λ*_max_ in chlorobenzene solution.

c Spin-coated from chlorobenzene solution onto a glass surface.

d *E*_HOMO_/*E*_LUMO_ = [−(*E*_onset_ − *E*_onset_ (FC/FC^+^*vs.* Ag/Ag^+^)) − 4.8] eV, where 4.8 eV is the energy level of ferrocene below the vacuum level and the formal potential *E*_onset_(FC/FC^+^*vs.* Ag/Ag^+^) is equal to 0.45 V.

e Electrochemical bandgap: E^el^_g_ = *E*_ox/onset_ − *E*_red/onset_.

f Optical bandgap: *E*g^opt^ = 1240/*λ*_edge_.

g Calculated by using Bragg's Law *d* = *λ*/(2 sin *θ*), where *λ* = 1.5406 Å. (—) Non-observable.

### Crystallinity and morphology

**Fig. 8:**
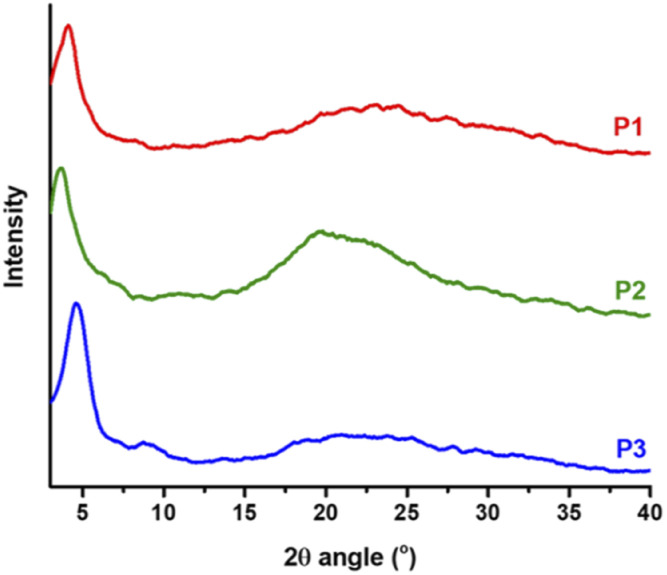
XRD patterns acquired for copolymers P1–P3.

**Fig. 9:**
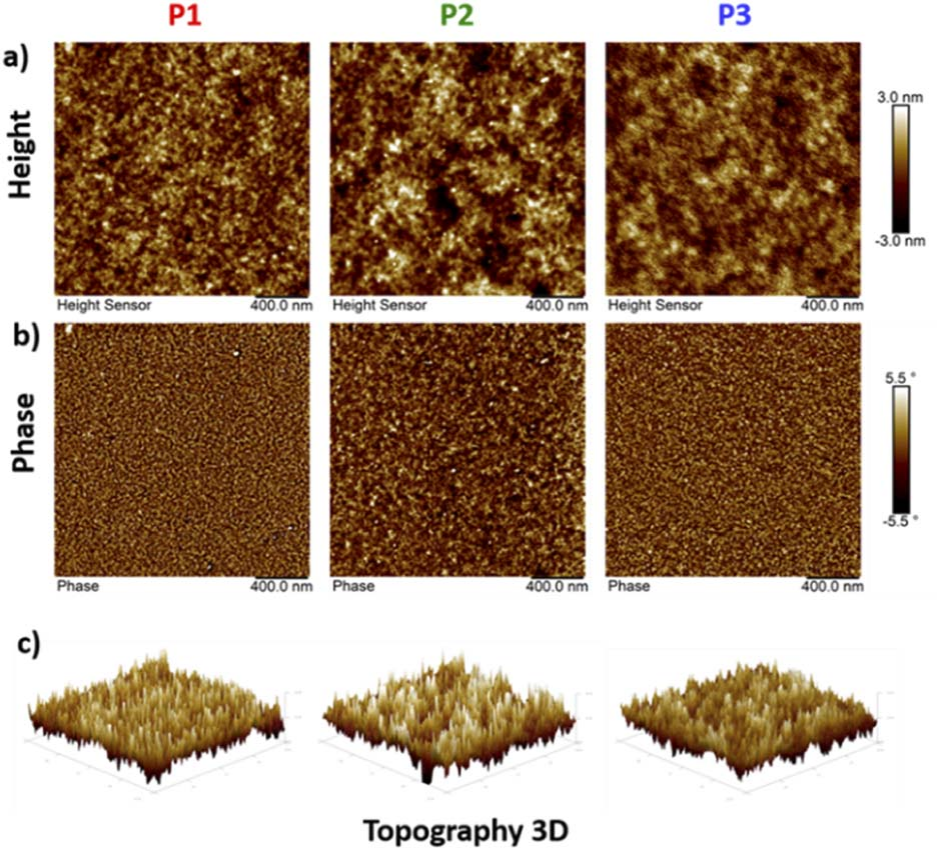
Tapping-mode AFM images of (a) the height, (b) phase with a 0.4 μm × 0.4 μm scan size and (c) 3D-topography of copolymers P1–P3.

Thin films of polymers P1–P3 were fabricated and measured using XRD in order to analyse the relationship between the chemical structure of the polymers and their crystallinity behaviour, and the lamellar/π–π stacking *d*-spacing values are summarized in Table 2. As depicted in Fig. 8, all polymers exhibit a similar degree of crystallinity with lamellar 2*θ* peaks in the range 3.7° to 4.6° and π–π stacking peaks ranging from 21° to 24°. With the branched side chains, P2 presented a larger lamellar packing distance (23.86 Å) compared to P1 (21.53 Å) and P3 (19.19 Å), which possess linear side chains. Similarly, the π–π stacking distance of P2 (4.50 Å) is also larger than that of P1 (3.77 Å) and P3 (3.69 Å). In addition, small lamellar reflection at 2*θ* = 7.2° was observed for P3. These results suggest that copolymer P3 presents better packing and higher crystallinity than homopolymers P1 and P2, which is in good alignment with the solid-state UV-vis absorption data and as a result could contribute to higher mobility performance in OTFT devices. Additionally, morphological properties in the solid-state thin films for P1–P3 were evaluated using atomic force microscopy (AFM). As shown in Fig. 9 the AFM images of P1 and P3 with linear side chains present slightly better homogeneity, in comparison with P2, which exhibits some nonuniform regions on the surface (Fig. 9a). P2 has a rougher surface with a root mean square (RMS) roughness of 1.04 nm, while P1 and P3 show slightly lower values of 0.80 and 0.75 nm, respectively. In addition, P2 presents bigger grain sizes as depicted in the phase image of Fig. 9b, in comparison with P1 and P3. These small differences were easily detected by 3D-height topography as shown in Fig. 9c (represented by the same scale for the polymers).

## Conclusion

In summary, we have reported the design, synthesis and characterization of four novel *S*,*N*-heteroacene based thiazole end-fused ladder monomers. Linear and branched alkyl groups on the nitrogen atoms afforded six fused aromatic ladder-type molecules M1 and M2. We further extended the conjugation length to afford ladder-type 5a–b with nine-fused rings in excellent solubility. In comparison to the thiophene-fused analogue SN6, the thiazole-fused molecules show a different molecular arrangement in the crystal structure and have a slightly enhanced coplanarity in the backbone. In addition, SN6-Tz exhibits highly structured and vibronically resolved absorption bands, which are red-shifted in comparison to the thiophene fused SN6 monomers exhibiting slightly lower absorption bands. Thus, replacing the thiophene units with the thiazoles in the ladder moiety slightly lowers the optical band gap from 3.12 eV to 3.03 eV. When increasing the conjugation length from six units in the thiazole ladder monomer to nine, the absorptions bands are significantly red shifted by 41–46 nm, thus further lowering the optical band gap. Furthermore, the thiazole-fused heteroacene SN6-Tz displayed a slightly higher Stokes shift compared to its thiophene fused SN6 counterpart.

Ladder homopolymers P1 and P2, and step-ladder copolymer P3 constructed from thiazole-fused *S*,*N*-heteroacene moieties present good solubility and high thermal stability up to 300 °C. All the polymers exhibit narrow band gaps ranging from 1.28 and 1.35 eV. The presence of the strong accepting DPP unit in the copolymer P3 backbone increases the π–π interactions and promotes the packing of polymer chains in the solid-state, which was investigated by thin-film absorption and XRD characterization. The absence of the DPP unit in the homopolymers P1 and P2 results in lower OTFT mobilities in the order of 10^−3^ and 10^−4^ cm^2^ V^−1^ s^−1^, respectively. Meanwhile P3 exhibits mobility values as high as 0.05 cm^2^ V^−1^ s^−1^. Overall, this study has demonstrated that step-ladder polymers consisting of thiazole-fused *S*,*N*-heteroacene building blocks can be suitable candidates for the fabrication of semiconductor OTFTs.

## Data availability

The datasets supporting this article have been uploaded as part of the ESI material.†

## Author contributions

S. Attar carried out the synthesis; R. Yang, Z. Chen & Y. Liu carried out OFET measurements; X. Ji did the DFT computational studies; M. Comi XRD and AFM measurements; S. Banerjee, and L. Fang edited and contributed in the writing of the manuscript with M. Al-Hashimi taking the led in with idea and finialzing.

## Conflicts of interest

There are no conflicts of interests to declare.

## Supplementary Material

SC-013-D2SC04661J-s001
